# Potential impacts of marine urbanization on benthic macrofaunal diversity

**DOI:** 10.1038/s41598-021-83597-z

**Published:** 2021-02-17

**Authors:** Kyosuke Momota, Shinya Hosokawa

**Affiliations:** 1 Central Laboratory, Marine Ecology Research Institute, 300 Iwawada, Onjuku, Isumi, Chiba 299-5105 Japan; 2 Port and Airport Research Institute, 3-1-1 Nagase, Yokosuka, Kanagawa 239-0826 Japan

**Keywords:** Biodiversity, Community ecology, Urban ecology, Biodiversity, Community ecology, Urban ecology

## Abstract

Urbanization and associated human activities have caused numerous changes to natural environments, including the loss of natural habitats and replacement with artificial structures. How these changes impact coastal marine biodiversity and ecosystem functioning is not well known. In this study, we examined the potential impacts of habitat changes by comparing species commonality and community structure (i.e., species richness, abundance, and functional composition) among artificial (a breakwater wall) and natural habitats (eelgrass bed, intertidal flat, and subtidal bottom) within a semi-enclosed coastal sea impacted by marine urbanization. We found considerable species overlap (i.e., high species sharing) among the eelgrass bed, intertidal flat, and subtidal bottom habitats. By contrast, the breakwater wall was a distinctive habitat with little overlap in species and functional groups with the other habitats, and was therefore a poor substitute for natural habitats. Our study suggests that marine urbanization degrades redundancy and inhibits the maintenance of biodiversity in coastal marine zones.

## Introduction

Urbanization entails dramatic environmental change and the expansion of artificial structures; this degrades biodiversity and ecosystem functioning (e.g., energy flow and nutrient cycles) by causing habitat loss, fragmentation, and homogenization^1–3^. Further urbanization in the future is inevitable given continued population growth and the proliferation of countermeasures against disasters associated with climate change^4–6^. In addition to the terrestrial realm, artificial structures are also expanding in prevalence in coastal marine zones (a process termed “marine urbanization”)^5,7,8^. Therefore, the impacts of marine urbanization and global population growth in coastal zones are matters of increasing concern^5,9^.

In coastal marine ecosystems, benthic macrofaunal communities are mainly composed of small invertebrates (e.g., crustaceans, polychaete worms, and molluscs), which are dominant mediators of material and energy flow^10,11^. Functional traits are one important factor in determining the distributions of these species, alongside other factors such as resource availability and habitat suitability^12–14^. For example, feeding traits (e.g., carnivory, detritivory, or filter feeding) can determine species distributions (in combination with resource availability) and shape their role as mediators. Also, life-form traits (e.g., free living, sedentary, or tube building) can be important depending on the available habitat characteristics. Therefore, habitat changes caused by marine urbanization can have varying effects on species distributions and community compositions depending on the functional traits of the species present. In turn, any resulting changes in functional group composition can further change the balance of ecosystem functions. While many benthic species inhabit marine artificial structures, their natural habitats, such as eelgrass beds and tidal flats, are important conservation targets that are currently under threat from various human activities (including marine urbanization) in many parts of the world^15,16^. Consequently, the impacts of marine urbanization on benthic macroinvertebrate communities cannot be ignored.

Like terrestrial urbanization, marine urbanization can cause major problems including habitat loss due to environmental alteration, as well as the replacement of natural habitats (e.g., seagrass beds, tidal flats, and rocky shores) with artificial structures (e.g., seawalls, piers, and pontoons). These changes can degrade ecosystem functioning by causing the replacement or loss of important taxa and/or functional diversity in marine environments^17–19^. The distinctive characteristics of artificial structures are of particular concern. First, artificial structures act as physical and environmental barriers to species and resources^20^. Second, these structures can facilitate the colonization of specific species (e.g., early-colonizing, opportunistic, and non-indigenous species) and thus support distinctive biological communities with low biodiversity^4,7,21–24^. Overall, in terms of species sharing, artificial structures are unlikely to establish redundant relationships with natural habitats, making them poor substitutes for natural habitats. Therefore, the replacement of natural habitats with artificial structures is likely to affect the biodiversity and functioning of coastal marine systems^15,25^. However, despite the accumulation of research on species distribution and biodiversity, studies focusing on functional diversity and ecosystem functioning remain scarce^26^.

In this study, we investigated potential habitat relationships (i.e., redundancy and uniqueness) by surveying the taxa and functional groups of benthic macrofaunal species present on four different habitat types (breakwater wall, eelgrass [*Zostera marina*] bed, intertidal flat, and subtidal bottom) in a semi-enclosed coastal sea affected by marine urbanization. Based on our research results, we discuss the expected impacts of marine urbanization on biodiversity. We surveyed community structures in periods of high (summer) and low (winter) productivity. Benthic macrofaunal communities vary seasonally in vegetated habitats such as seagrass beds^27,28^. Therefore, we conducted our observations during these stable seasonal extremes to reveal the range of relationships between vegetated and unvegetated habitats.

## Methods

### Study area

**Figure 1:**
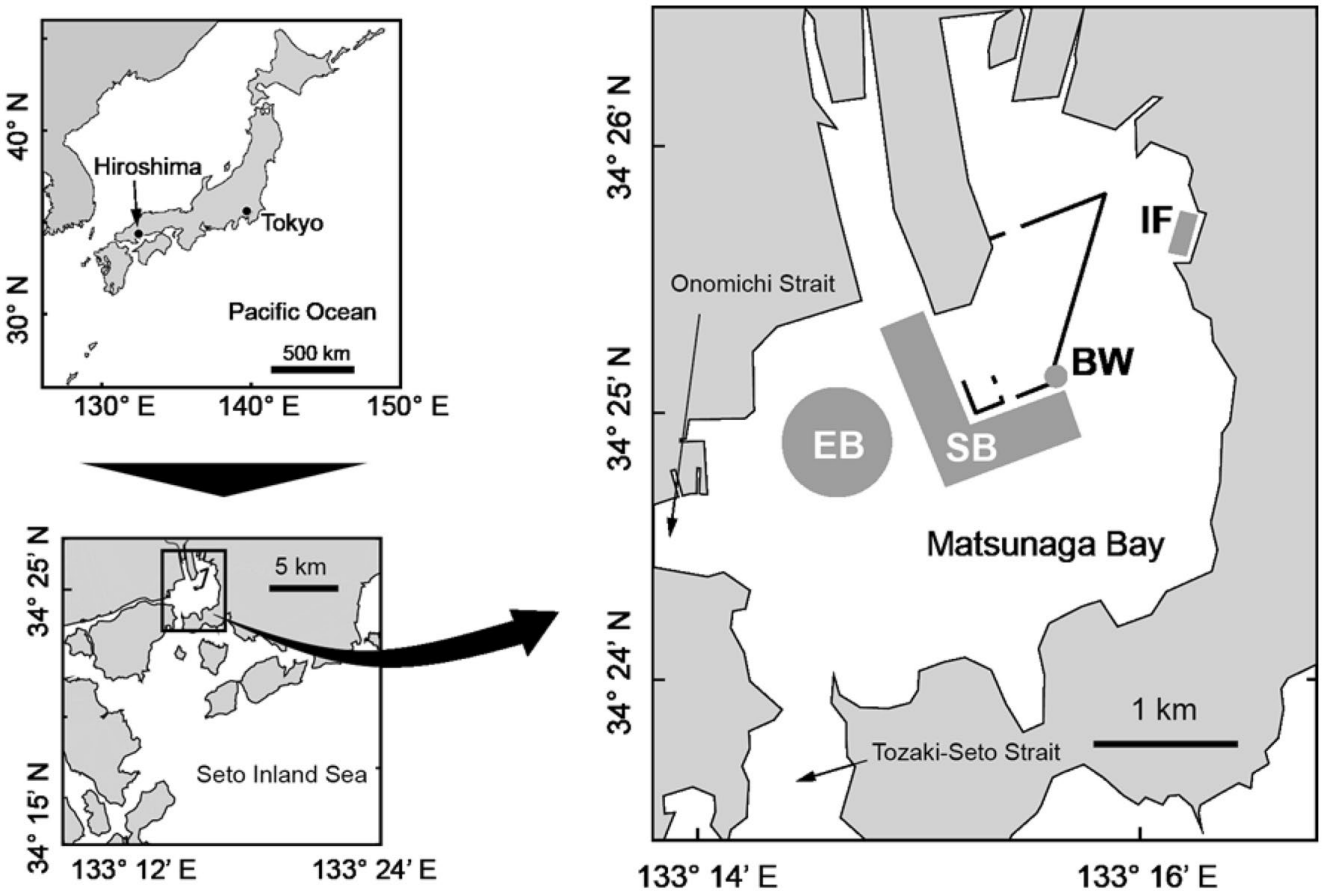
Locations of sampling sites in Matsunaga Bay, Hiroshima, Japan. This map was created based on coordinate data from Google (http://www.gis-tool.com/mapview/maptocoordinates.html). The four habitats examined in this study are indicated by BW (breakwater wall; grey circle), EB (eelgrass bed; grey circle), IF (intertidal flat; grey rectangle), and SB (subtidal bottom; grey polygon).

We established field survey sites in four different habitat types (BW: breakwater wall; EB: eelgrass bed; IF: intertidal flat; SB: subtidal bottom) within Matsunaga Bay, located in the eastern Seto Inland Sea, Japan (Fig. 1). Matsunaga Bay is a small semi-closed bay with an area of approximately 12 km^2^. It is connected with other water bodies through the Tozaki-Seto Strait (width: approx. 400 m) and the Onomichi Strait (width: approx. 200 m)^29,30^. Water depths are mostly less than 20 m throughout the bay. The water depths at our four survey sites were approximately 4.5 m, but part of site SB located near a shipping channel reached depths of 10–13 m. Intertidal flats cover 35% (4.3 km^2^) of the bay area, whereas eelgrass beds cover 1.7% (0.2 km^2^)^31^. The bottom sediment type is mainly muddy throughout the bay, although some parts of EB and SB have sandy and muddy bottoms (see Supplementary Table S1)^30^.

Although human activities along the coast of Matsunaga Bay appear to be associated with artificial structures (e.g., industrial plants, marinas, and lumber yards), natural habitats are still relatively well-preserved compared to areas in the eastern Seto Inland Sea^29^. The total population of towns within 5 km of the coastline of the bay can be expected to exceed 100,000 people, which ranks within the top 20% of administrative districts in Japan, including prefectures, towns, villages, and the 23 wards of Tokyo^32^.

### Data collection

We conducted one field survey in September 2016 (summer) and another in January 2017 (winter) to collect data on benthic community structures and environmental conditions in each habitat. We established five sampling sites within each habitat to obtain replicated samples. To reduce biases due to tidal cycle, we performed all field sampling and measurements at high tide, when all habitats were underwater.

We used a standard sample area (approximately 0.15 m^2^ per sample) at each sampling site irrespective of sampling method to obtain comparable data on benthic communities. Some of our sampling methods involved Smith–McIntyre grab samplers and quadrats that could not cover the standard sample area in a single sample; for these methods, we combined the data from three samples to make up a single sampling site. At BW, benthic macrofauna (hereafter referred to as “benthos”) samples were collected by SCUBA divers. We established 15 sampling positions in a 5 × 3 grid (i.e., five depths [sampling sites] and three replicates) based on distance from the seafloor at the breakwater wall (Supplementary Fig. S1). The SCUBA divers used scraper blades, 0.1-mm mesh bags, and 22.5 cm × 22.5 cm quadrats because the benthic communities were mainly composed of sessile organisms. At EB, we employed different sampling methods for the above- and belowground components. SCUBA divers collected aboveground samples of eelgrass-associated benthos and eelgrass shoots using a mesh bag (mesh size: 0.1 mm; bag diameter: 45 cm). They then cut away the eelgrass shoots near each aboveground site and collected belowground samples of the benthos on top of and within the sediment by using the bucket part of a Smith–McIntyre grab sampler (sampling area: 22.5 cm × 22.5 cm). At IF and SB, benthos and bottom sediment samples were collected from a ship using a Smith–McIntyre grab sampler (sampling area: 22.5 cm × 22.5 cm).

All benthos was extracted using a 1-mm sieve and preserved in buffered 10% formalin in the field as soon as possible after sampling. The samples were identified to the lowest possible taxonomic unit and counted in the laboratory. After identification, we organized the dominant benthic species (or taxa) according to their primary feeding types and common life forms with reference to the World Register of Marine Species (http://www.marinespecies.org/) and the literature. No vertebrate species were targeted in this study. We defined the primary life forms of adult benthic species on/in their substrates as “common life forms.”

Although differences in environmental conditions were not the focus of our study, we did assess whether there were considerable water quality differences among sites. The purpose of this assessment was to try to identify sites with similar conditions so that exogenous impacts on biological communities could be discounted as much as possible in the analysis. We measured water and sediment conditions at each sampling site (except at BW, where sediment conditions were not measured due to the absence of sediment). Prior to benthos and sediment sampling, we measured water temperature, salinity, pH, and dissolved oxygen concentration at each site at a depth directly above the seafloor by using a multi-parameter water quality meter (AAQ‐RINKO, JFE Advantech Co. Ltd., Japan). At BW, where the substrate (i.e., the breakwater) is oriented vertically (see Supplementary Fig. S1), we measured water conditions in the middle of the water column. We also measured temperature, pH, oxidation–reduction potential (ORP), water content, and median particle size (D_50_) in the sediment. Sediment temperature, pH, and ORP were measured by using a portable ion meter (IM-32P, DKK-TOA Co., Japan) immediately after each sample was collected. Sediment water content and D_50_ were measured in the laboratory once benthic species had been removed from the sample.

### Data analysis

First, we identified how many species were shared between all habitat pairs to understand inter-habitat species-sharing relationships. Second, we compared species compositions and abundances among habitats using similarity indices and multivariate analysis (described below).

To detect species sharing in terms of species commonality and endemism among the four habitats, we classified benthic species into the following three categories: common, endemic, and shifting. Common species were defined as species that occurred across all habitats. Endemic species are those that were found in only one habitat. Shifting species were defined as those that occurred in multiple habitats (but not across all habitats) and therefore showed a broad allowable range of habitat types or conditions. To analyse the importance of habitat sets in maintaining local species diversity, we further categorized the shifting species into two- or three-habitat users (i.e., those that occurred in two or three different habitats). Moreover, we calculated the numbers and proportions (i.e., using the Jaccard similarity index) of shared species in each habitat pair to evaluate the potential strength of any inter-habitat relationships. The Jaccard similarity index ($J$) is calculated as follows:

$$ J = \frac{{S_{\alpha \beta } }}{{S_{\alpha } + S_{\beta } - S_{\alpha \beta } }}, $$

where $S_{\alpha }$ is the number of species in habitat $\alpha$, $S_{\beta }$ is the number of species in habitat $\beta$, and $S_{\alpha \beta }$ is the number of species that are shared among habitats $\alpha$ and $\beta$.

In terms of the functional groups, we analysed abundance matrices of abundant species grouped by primary feeding types and common life forms. Focusing on abundant species is a useful way to reflect the functional characteristics of biological communities^14^. Therefore, we identified the most abundant species from each sample before constructing the abundance data matrices. To determine how many species to select for analysis, we calculated the number *e* of equally-abundant species that would be required to obtain the Simpson diversity index of each community (i.e., the effective number of species^33^). We then selected *e* abundant species from each sample in rank order from most to least abundant. If multiple species of the same rank occupied this cut-off threshold, we selected all of them. This selection method, which is unique to our study, successfully identified dominant species that accounted for over 70% of the total abundance in each community in all habitats.

To compare benthic macrofaunal communities among habitats, we performed non‐metric multidimensional scaling (NMDS)^34^ based on dissimilarity matrices obtained by using the *metaMDS* function in the *vegan* package^35^. To compare species compositions, we constructed Jaccard dissimilarity matrices based on presence/absence data, and to compare species abundance and functional compositions, we constructed Bray–Curtis dissimilarity matrices based on abundance datasets. All abundance datasets were square-root transformed before calculating the Bray–Curtis dissimilarity matrices to reduce the influence of abundance bias. We accepted the NMDS ordinations if stress values were less than 0.2 to maintain the accuracy of the two‐dimensional ordinations^34^. Then, we tested the effects of habitat type and sampling time by conducting a two-way permutational multivariate analysis of variance (two-way PERMANOVA)^36^ using the *adonis* function in the *vegan* package. Here, we considered four habitats (Habitat), two sampling times (Time), and their interaction term as explanatory factors. Although our main focus was differences in community compositions among habitats at each sampling time, we also examined the magnitude of variation in each habitat by comparing two stable seasonal extremes (i.e., summer and winter). If the results of the PERMANOVA were significant, we performed post-hoc tests (pairwise PERMANOVA) to identify which pairs of community structures were significantly different by using the *pairwise.perm.manova* function in the *RVAideMemoire* package^37^. We used 9999 permutations for the NMDS ordination, PERMANOVA, and pairwise PERMANOVA. *P*-values calculated during the pairwise PERMANOVA were corrected using the false discovery rate method^38^. For the benthic community data collected at each sampling point at EB, above- and belowground datasets were integrated to reflect spatial representativeness (see Figs. S3 and S4). All analyses were performed using R version 3.5.1^39^.

## Results

### Environmental conditions

Water conditions were largely homogeneous across habitats at each sampling time (Supplementary Table S1), although wintertime water temperatures at IF were 2–3 °C lower on average than in the other habitats. Dissolved oxygen concentrations were within the range 4.9–9.3 mg/L, and sediment pH and ORP were similar across all habitats and sampling times. Sediment temperatures at IF were slightly higher than at the other habitats in summer but 2–3 °C lower in winter. Sediment water content was the highest at SB, followed by EB and IF, regardless of sampling time.

### Species commonality and endemism

We collected a total of 256 benthic macrofaunal species during this study (see Supplementary Table S2 for details), with 181 species collected during summer and 196 species during winter (Table 1). The number of species collected was especially high at BW (summer: 87 species; winter 98 species) and EB (summer: 88; winter: 77) (see Supplementary Fig. S2 for mean values at each site). IF had more moderate species richness, with 54 species collected in summer and 65 in winter. SB had the lowest number of species in both summer (39 species) and winter (38 species). BW and EB consistently contained about 40–50% of the total species observed.

**Table 1:** The number of common, endemic, and shifting species in each habitat.

Category	BW	EB	IF	SB	*ALL*
**SUMMER**					
Common species	6 (6.9%)	6 (6.8%)	6 (11.1%)	6 (15.4%)	***6 (3.3%)***
Endemic species	62 (71.3%)	37 (42.0%)	15 (27.8%)	9 (23.1%)	***123 (68.0%)***
Shifting species	19 (21.8%)	45 (51.1%)	33 (61.1%)	24 (61.5%)	***52 (28.7%)***
***Total***	***87 (48.1%)***	***88 (48.6%)***	***54 (29.8%)***	***39 (21.5%)***	***181***
**WINTER**					
Common species	6 (6.1%)	6 (7.8%)	6 (9.2%)	6 (15.8%)	***6 (3.1%)***
Endemic species	83 (84.7%)	31 (40.3%)	24 (36.9%)	5 (13.2%)	***143 (73.0%)***
Shifting species	9 (9.2%)	40 (51.9%)	35 (53.8%)	27 (71.1%)	***47 (24.0%)***
***Total***	***98 (50.0%)***	***77 (39.3%)***	***65 (33.2%)***	***38 (19.4%)***	***196***

Numbers in parentheses for the three species groups show species percentages relative to the total number of species in each habitat. Numbers in parentheses in rows marked ***Total*** are percentages relative to the total number of species across all habitats in each sampling time. Habitat abbreviations are as shown in Fig. 1.

Common species made up 3.5% (9/256) of the total number of species observed (Table 1), and only three species were consistently observed across all habitats during both sampling times; namely, the Asian mussel *Arcuatula senhousia*, the polychaete worm Hesionidae sp., and the ribbon worm Nemertea sp. (Supplementary Table S2). Six additional species were observed across all habitats during one sampling time but not the other. In summer, these were the gastropod *Reticunassa festiva* and the polychaete worms Capitellidae sp. and *Scolelepis* sp. In winter, these were the polychaete worms *Eulalia viridis*, *Pseudopolydora* sp., and Sabellidae sp.

Endemic species made up 68.0% (123/181) of the total species in summer and 73.0% (143/196) in winter (Table 1). Among habitats, endemism was highest at BW (summer: 71.3%; winter: 84.7%), followed by EB (42.0%; 40.3%), IF (27.8%; 36.9%), and SB (23.1%; 13.2%) during both sampling times.

**Figure 2:**
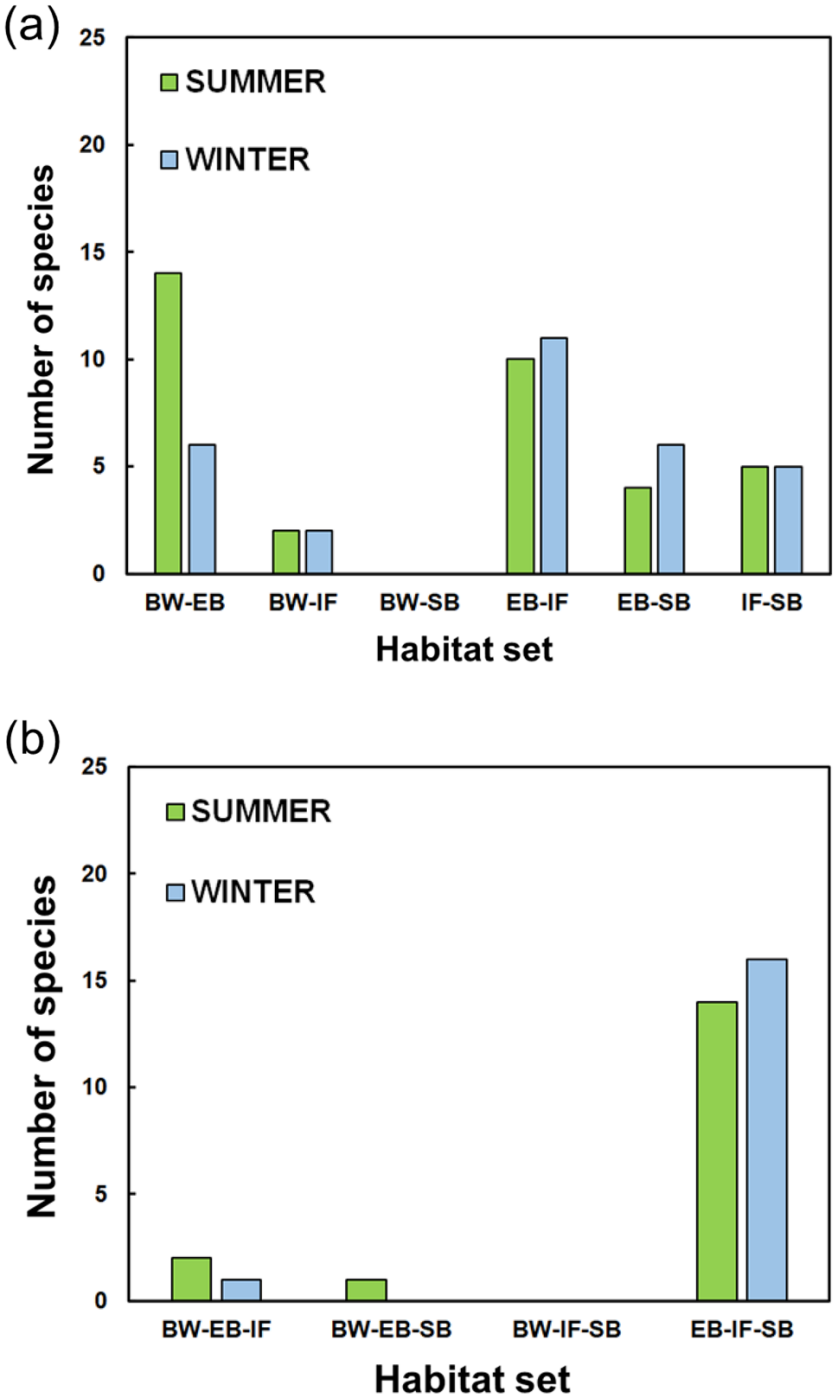
Numbers of shifting species in each examined habitat. Panels show (**a**) two-habitat users and (**b**) three-habitat users. Habitat abbreviations are as shown in Fig. 1.

**Figure 3:**
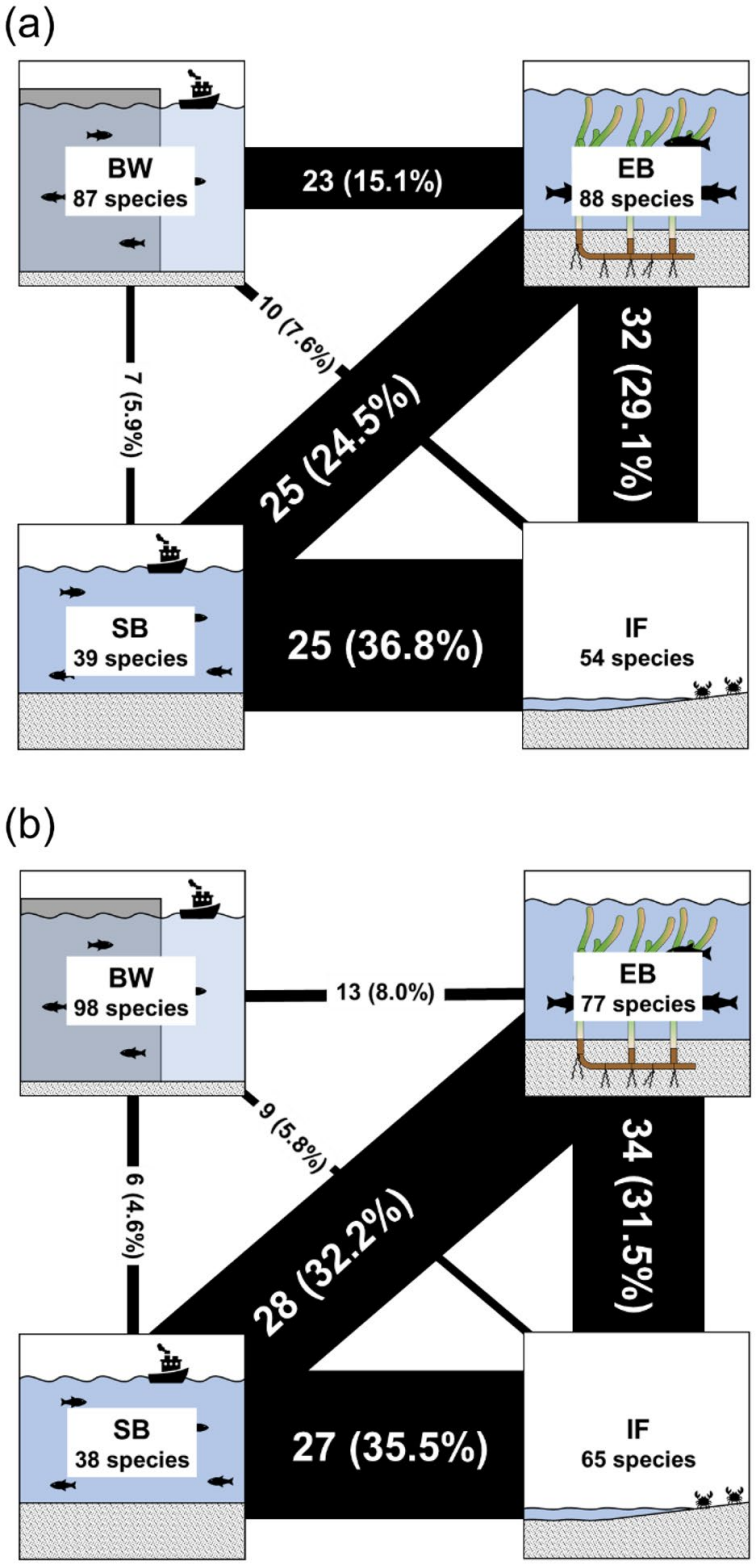
Shared species in habitat pairs. Panels show data for (**a**) summer and (**b**) winter. Black numbers in each box indicate total numbers of species collected in each habitat. White or black numbers on the black lines indicate the numbers of species that were shared between each habitat pair with percentages based on Jaccard indices shown in parentheses. The thickness of each black line is proportional to the percentage of species shared. Habitat abbreviations are as shown in Fig. 1.

Shifting species made up 28.7% (52/181) of the total species in summer and 24.0% (47/196) in winter (Table 1). During both sampling times, over 85% of shifting species were observed at EB. At IF and SB, we identified approximately 30 shifting species in summer and winter. We identified 35 two-habitat users and 17 three-habitat users in summer and 30 two-habitat users and 17 three-habitats users in winter. BW shared a few shifting species with IF and SB (Fig. 2). The highest number of two-habitat users were shared between BW and EB, meaning that many of the shifting species observed at BW were also identified at EB. The highest number of three-habitat users were shared among EB, IF, and SB during both sampling times. Similarly, the total number of shared species was relatively high among EB, IF, and SB regardless of sampling time (Fig. 3). The species shared between BW and SB were mainly common species (see Table 1; Fig. 3).

### Species composition and abundance

**Figure 4:**
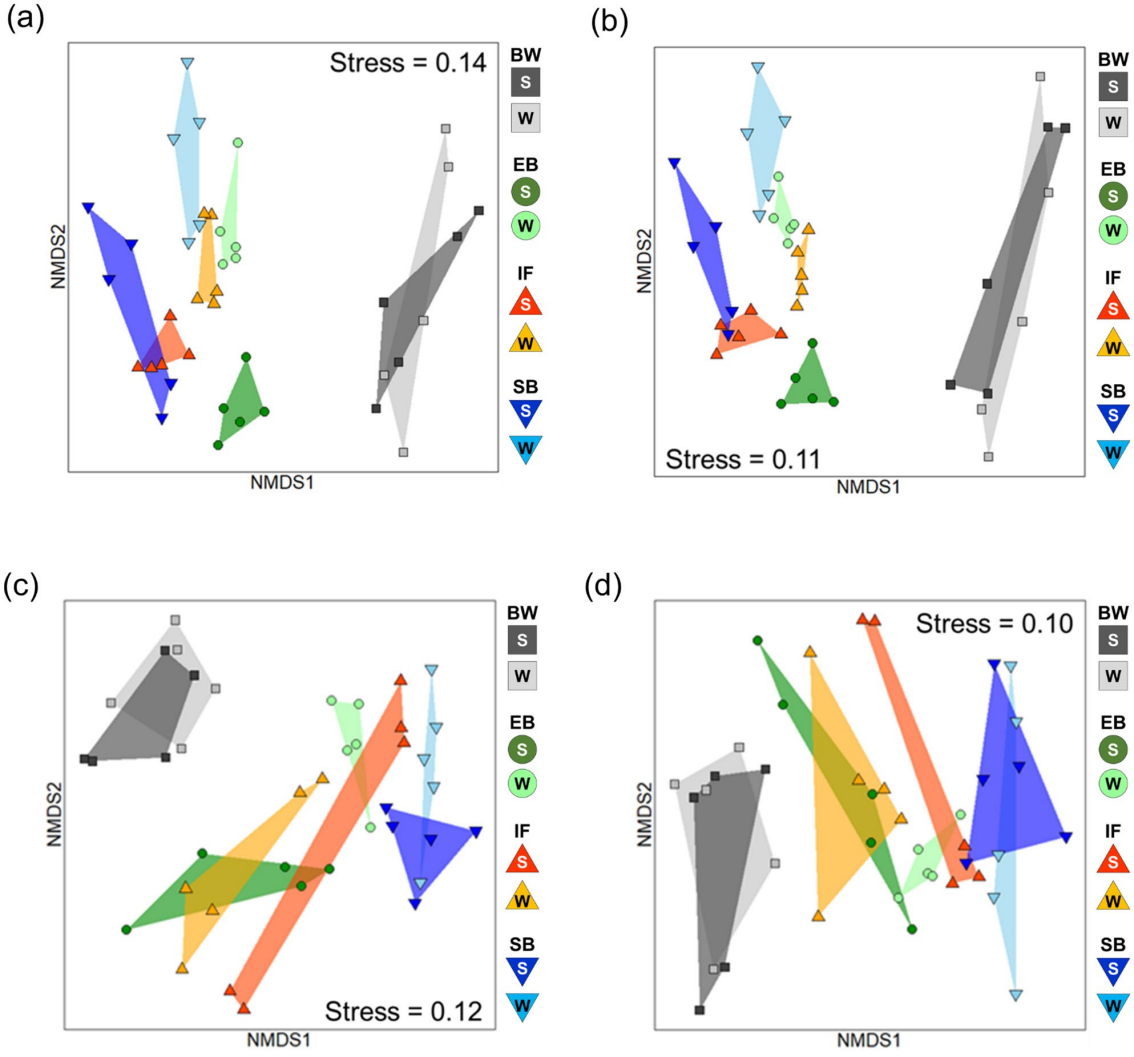
Non‐metric multidimensional scaling (NMDS) ordinations of (**a**) species compositions, (**b**) species compositions and abundances, (**c**) abundance compositions based on primary feeding type, and (**d**) abundance compositions based on common life forms. The four different symbols indicate subsites in each habitat. Convex hulls enclosed by symbols indicate the dispersion of community composition within habitats. Habitat abbreviations are as shown in Fig. 1.

Patterns in total benthic species abundance per unit area at each habitat differed considerably from the distribution of species richness (Supplementary Fig. S2). BW had by far the highest total abundance among habitats in our study during both sampling times. EB and IF had contrasting temporal trends, but similar overall average abundances. SB had the lowest average abundance during both sampling times. NMDS analysis of species composition and abundance revealed similar patterns among habitats (Fig. 4a,b). PERMANOVA results show that these variations in species composition were much more strongly explained by habitat than by sampling time (Table 2). Species compositions and abundance at BW were considerably different from those at EB, IF, and SB. In particular, BW and SB were the most different of any habitat pair. Pairwise habitat comparisons showed that all habitat pairs were significantly different from each other in both summer and winter (Table 3). However, summertime species compositions and abundances at BW were not significantly different from wintertime values.

**Table 2:** Results of permutational multivariate analysis of variance testing for differences in benthic community compositions.

Factor	df	SS	MS	*F*	*R*^*2*^	*P* (> F)
**Community composition**						
Species composition						
Habitat	3	4.34	1.45	5.65	0.286	***0.0001***
Time	1	1.03	1.03	4.04	0.068	***0.0001***
Habitat × time	3	1.59	0.53	2.07	0.105	***0.0001***
Residuals	32	8.19	0.26		0.541	
Abundance composition						
Habitat	3	5.18	1.73	8.55	0.360	***0.0001***
Time	1	1.11	1.11	5.48	0.077	***0.0001***
Habitat × time	3	1.63	0.54	2.70	0.114	***0.0001***
Residuals	32	6.46	0.20		0.449	
**Functional composition**						
Primary feeding type						
Habitat	3	4.39	1.46	17.40	0.526	***0.0001***
Time	1	0.23	0.23	2.71	0.027	***0.0423***
Habitat × time	3	1.03	0.34	4.09	0.124	***0.0003***
Residuals	32	2.69	0.08		0.323	
Common life form						
Habitat	3	3.78	1.26	17.38	0.565	***0.0001***
Time	1	0.05	0.05	0.64	0.007	0.5933
Habitat × time	3	0.54	0.18	2.50	0.081	***0.0225***
Residuals	32	2.32	0.07		0.347	

Significant *P*-values (*P* < 0.05) are shown in bold italic typeface.

**Table 3:**
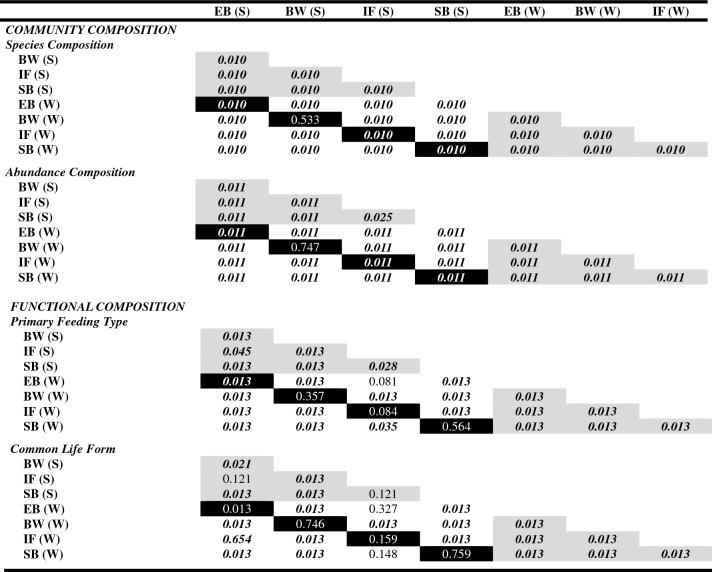
*P-*values obtained from post-hoc tests (pairwise PERMANOVAs) testing for similarities in community and functional composition across habitats and sampling times (S: summer; W: winter).

Significant *P*-values (*P* < 0.05) are shown in bold italic typeface. Grey cells show comparisons among habitats within sampling times. Black cells show comparisons between sampling times for each habitat. Habitat abbreviations are as shown in Fig. 1.

### Functional composition

**Figure 5:**
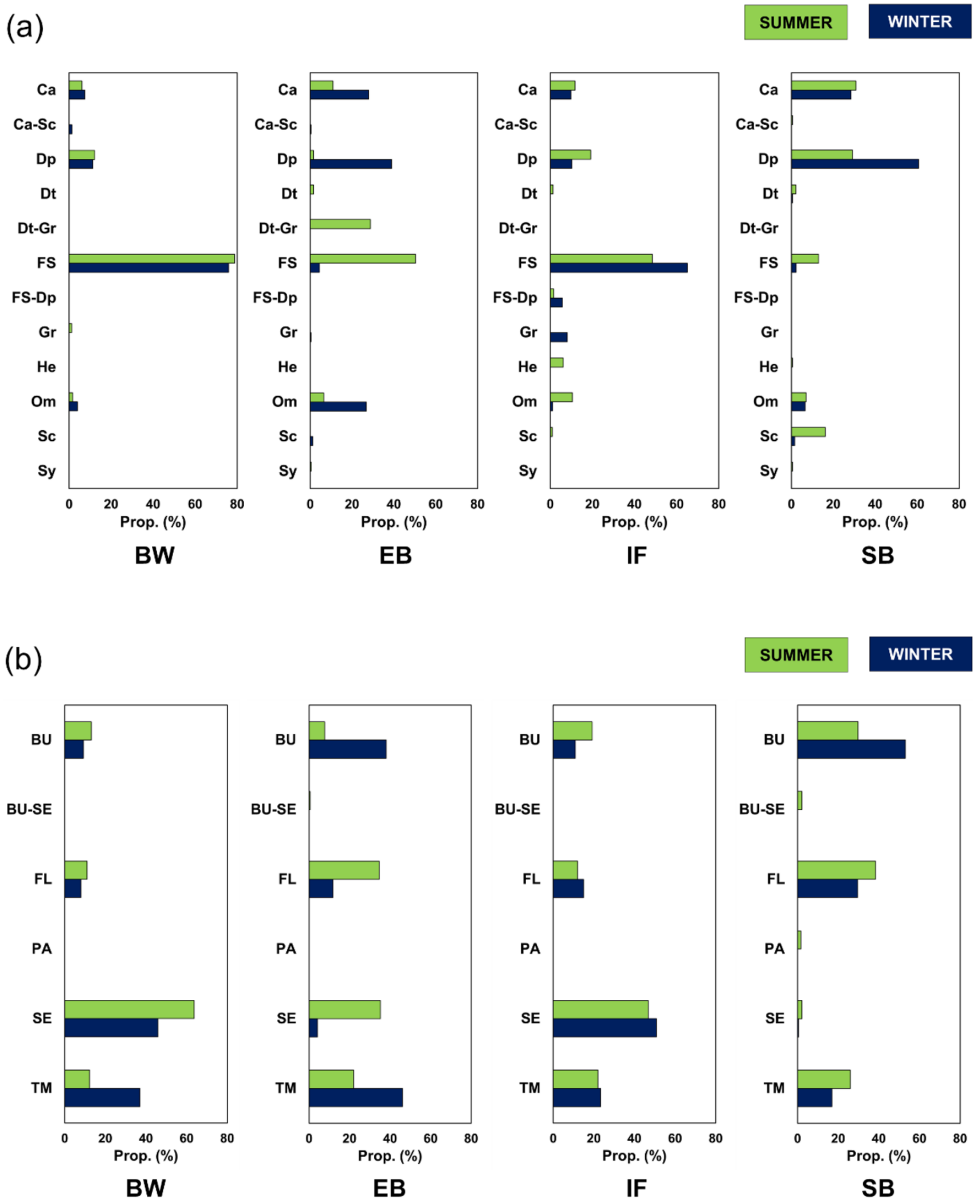
Relative abundance of (**a**) primary feeding types and (**b**) common life forms. Functional groups are abbreviated as follows: Primary feeding types: *Ca* carnivore, *Dp* deposit feeder, *Dt* detritus feeder, *FS* filter/suspension feeder, *Gr* grazer, *He* herbivore, *Om* omnivore, *Sc* scavenger, *Sy* symbiont. Common life forms: *BU* burrowing, *FL* free-living, *PA* parasite, *SE* sedentary, *TM* tube/mucus-sheath building. Habitat abbreviations are as shown in Fig. 1.

The abundant species observed in our study were classified into 12 primary feeding types and 6 common life forms (Fig. 5; see Supplementary Table S2). According to the NMDS results, variations in both types of functional composition are mainly explained by habitat differences (Fig. 4c,d). These were also supported by the PERMANOVA results (Table 2). Notably, habitat differences had a greater influence on functional compositions than on species compositions and abundances regardless of sampling time. The magnitude of any sampling time effect was small and similar to those of species compositions and abundances.

Habitat differences contributed to 52.6% of the variation in primary feeding types (see *R*^2^ values in Table 2). Pairwise comparisons revealed that feeding type compositions differed significantly for all habitat pairs during both sampling times (Table 3). Unlike with species compositions and abundances, temporal differences in feeding type composition were only observed at EB. EB was dominated by detritivores and filter and/or suspension feeders in summer and by deposit feeders and omnivores in winter (Fig. 5a). At BW, filter and/or suspension feeders were dominant and accounted for over 75% of the species. Although these feeding types were also dominant at IF, more diverse functional groups were also present. Carnivores and deposit feeders dominated at SB.

Habitat differences contributed to 56.5% of the variation in common life forms (see *R*^2^ values in Table 2). Pairwise comparisons revealed that life form compositions differed significantly among nearly all habitat pairs during both sampling times (Table 3). Life form compositions at IF, however, were not significantly different from those at EB and SB in summer (Table 3). As with the primary feeding types, temporal differences in common life form composition were only observed at EB. Free-living and sedentary species dominated at EB in summer, while burrowers and tube/mucus sheath builders were dominant in winter (Fig. 5b). Although sedentary species dominated at BW and IF during both sampling times, the degree of dominance differed among the two habitats. Unlike in the other three habitats, life form compositions at SB were dominated by burrowers, free-living species, and tube/mucus sheath builders during both sampling times.

## Discussion

Our results show different interrelationships in terms of shared species (or taxa) and functional groups among four benthic macrofaunal habitats in semi-enclosed coastal waters affected by marine urbanization. In terms of species sharing, we found clear differences between the role of BW and the three natural habitats (EB, IF, and SB) during both sampling times. Among the three natural habitats, approximately 30% of species were shared, and certain redundant relationships, which contribute to the maintenance of the species with each other, were established. By contrast, the breakwater wall featured a distinctive community during both sampling times.

The similarity of the substrates in the three natural habitats could explain the species-sharing relationships among them. We separated the community at EB into eelgrass shoot (aboveground) and muddy sand bottom (belowground) components and found that the aboveground community shared much fewer species with IF and SB than the belowground community (Supplementary Fig. S3). Furthermore, the aboveground species composition differed significantly from those at IF and SB, whereas the belowground species composition was similar to those at IF and SB (Supplementary Fig. S4a). Polychaete burrowers, which prefer soft sediments, were the main taxa shared among the three habitats, which all featured sand and mud as the primary substrates (Supplementary Tables S1, S2).

The different degrees of species-sharing in the above- and belowground communities imply that environmental complexity within eelgrass beds creates redundant relationships with unvegetated habitats (i.e., IF and SB) and enhances the capacity of the habitat to host a variety of species. Aboveground seagrass structures act as secondary substrates for epibiotic organisms, thereby boosting species diversity^20,40,41^. As shown in our results, the aboveground seagrass structures appear to support unique benthic macrofaunal communities (Supplementary Fig. S4). Although belowground structures formed by seagrass roots and rhizomes do not always enhance benthic species diversity^42,43^, the synergistic effects of the above- and belowground components can enhance diversity of both epifauna and infauna through detritus accumulation^44^. Variable environments allow diverse species to coexist with minimal niche overlap because niche diversification is easily promoted by the many opportunities for resource partitioning^45^. The results of our functional group analysis also support this mechanism for maintaining diversity. Functional groups favouring aboveground resources (i.e., detritus feeders, grazers, and filter/suspension feeders) and structures (i.e., free-living, sedentary) predominated in summer when aboveground eelgrass biomass was high. In contrast, functional groups favouring sediment and/or sediment-related resources (i.e., deposit feeders) and structures (i.e., burrowing and tube/mucus-sheath building) predominated in winter when aboveground eelgrass biomass was low (Supplementary Table S1, Fig. 5). Thus, vegetated habitats (i.e., variable environments) are presumably able to create more species- and functional-group-sharing paths than unvegetated habitats (i.e., single-substrate habitats with relatively stable environments) in a habitat network under similar environmental conditions.

The dominant functional groups (e.g., sedentary filter and/or suspension feeders and free-living gastropods) at BW, an artificial hard-substrate habitat, are also dominant in natural hard-substrate habitats (e.g., rocky shores and reefs) in temperate regions of Japan^46,47^. However, studies in other Japanese temperate habitats show that natural and artificial hard-substrate habitats that share similar physical and chemical environments do not always develop the same benthic macrofaunal communities^48,49^. Construction materials and the location and timing of construction appear to influence the formation of communities in artificial habitats by disrupting species life cycles and changing how various species interact. As a result, species commonality and similarity in functional group composition are not always high among natural and artificial hard-substrate habitats.

Interestingly, the species and functional group compositions did not necessarily appear to change synchronously. These compositional changes were synchronized in BW and EB, but not in IF and SB (Table 3, Fig. 5). This asynchronous change in IF and SB suggests a mechanism facilitating the acceptance of specific functional groups in habitats with low substrate variability regardless of temporal species turnover. Focusing on primary feeding type, the compositions of all habitats differed from each other at both sampling times, and there were no significant differences between the two sampling times for all three habitats except EB (Table 3; Figs. 4c, 5a); these results imply that each benthic community is functionally unique. Although our results are consistent with the effects of habitat diversity losses identified in previous research^17,18^, our study additionally shows that functional diversity may be more quickly and critically affected than species diversity.

Our study suggests that marine urbanization and its accompanying natural habitat losses and incursion of marine artificial structures degrades biodiversity in coastal marine zones. Maintaining and enhancing biodiversity may require the maintenance of environmentally variable habitats and high habitat diversity at the substrate level. Crucially, artificial habitats cannot be expected to substitute for natural habitats. Although the communities in BW and IF appear to be functionally similar as they are mainly composed of filter/suspension feeders (Fig. 5a), quantitative comparisons in the field are required to compare their true performance^26^. As the roles of artificial habitats have become a matter of increasing concern, coastal development based on eco-engineering has attracted recent attention^25^. For example, biodiversity-conscious construction based on the concept of green infrastructure seeks to enhance marine artificial structures through the addition of water retention functionality, micro-spaces created by pits and grooves, and foundation species (e.g., seaweeds and corals)^50–52^. However, such designed structures can prove inefficient or even detrimental unless compatibility with surrounding environments is sufficiently considered during the design stage^25^. Therefore, a deeper understanding of how species and functional groups are shared among various habitats, including those outside the scope of this study, is essential to inform future countermeasures against marine urbanization.

## Supplementary Information

Supplementary Information.

## Data Availability

All data generated or analysed during this study are included in this published article and in the Supplementary information.
