# Supplementary material for: Potential impacts of marine urbanization on benthic macrofaunal diversity
Source: Sci Rep. 2021 Feb 17;11:4028. doi: 10.1038/s41598-021-83597-z (PMC7889940; doi:10.1038/s41598-021-83597-z)
Supplement: Supplementary file 1 — Supplementary Information. [file 41598_2021_83597_MOESM1_ESM.pdf]

Potential impacts of marine urbanization on benthic macrofaunal diversity

- Supplementary data -

Kyosuke Momota[1,2,*] and Shinya Hosokawa[2]

[1] Central Laboratory, Marine Ecology Research Institute, 300 Iwawada, Onjuku, Isumi, Chiba 299-5105, Japan

[2] Port and Airport Research Institute, 3-1-1 Nagase, Yokosuka, Kanagawa 239-0826, Japan.

*Corresponding author

Supplementary figures

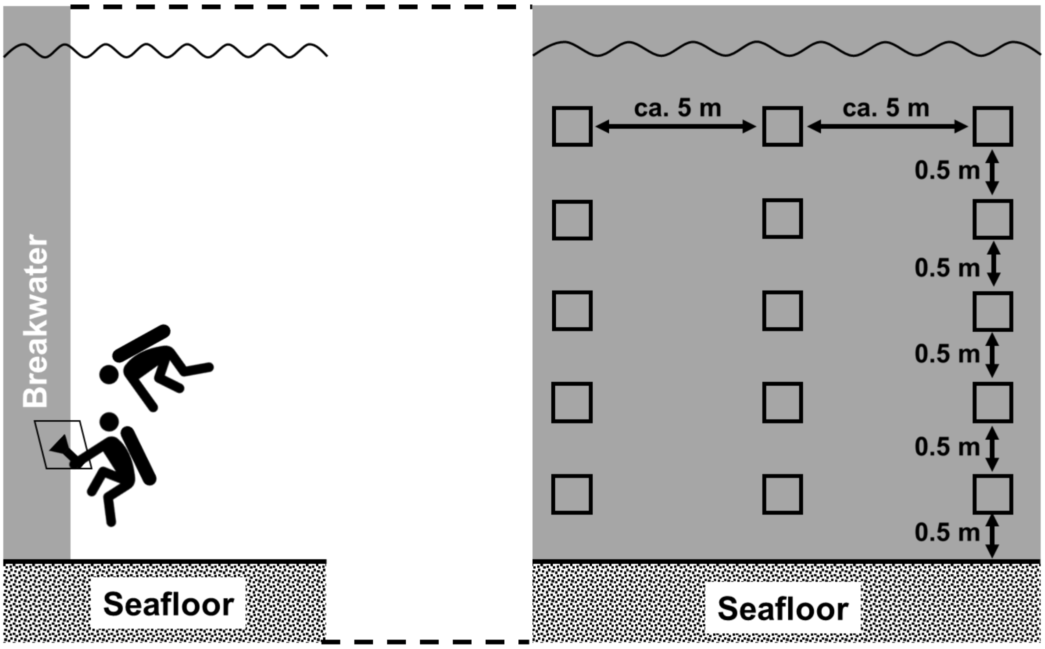

Figure S1. Schematic diagram showing the arrangement of sampling points (unfilled squares) at the breakwater wall habitat. The left panel shows a cross-sectional view and the right panel a plan view of the breakwater wall.

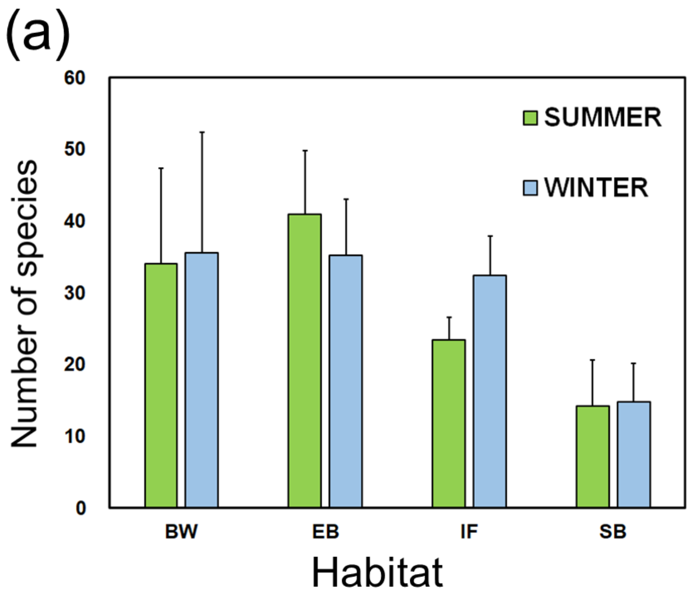

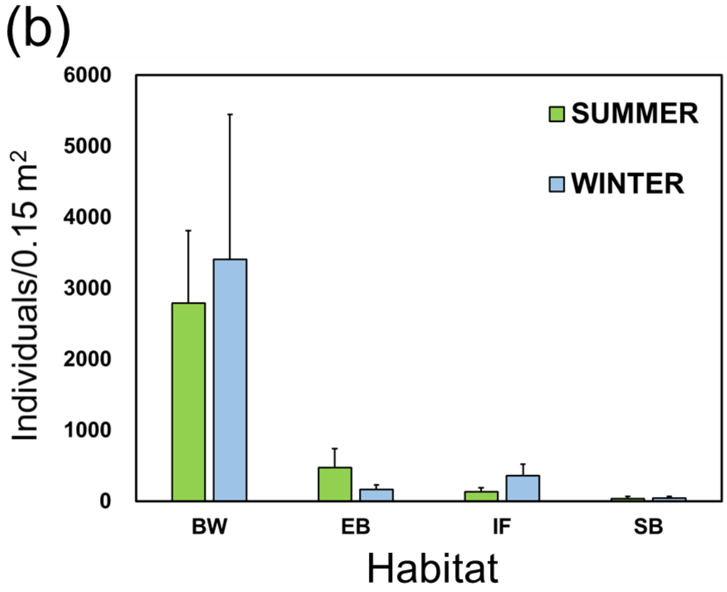

Figure S2. Mean (a) species richness and (b) total abundances at each habitat. Error bars show standard deviations. Habitat abbreviations are as in Fig. 1.

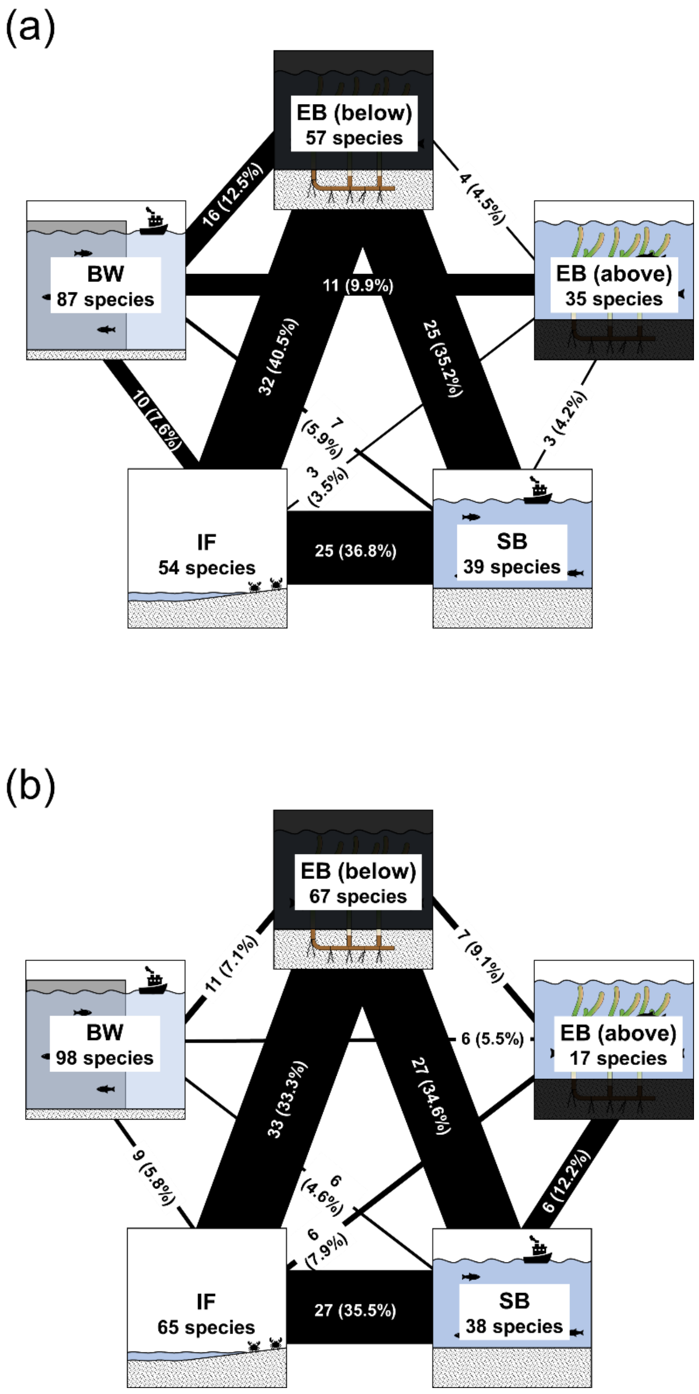

Figure S3. Shared species among habitat pairs in (a) summer and (b) winter. The eelgrass bed (EB) was separated into above- and belowground communities. Black numbers in each box indicate total numbers of species collected in each habitat. White or black

numbers on the black lines indicate the numbers of species that were shared between each habitat pair, with percentages based on Jaccard indices shown in parentheses. The thickness of each black line is proportional to the percentage of species shared. Habitat abbreviations are as shown in Fig. 1.

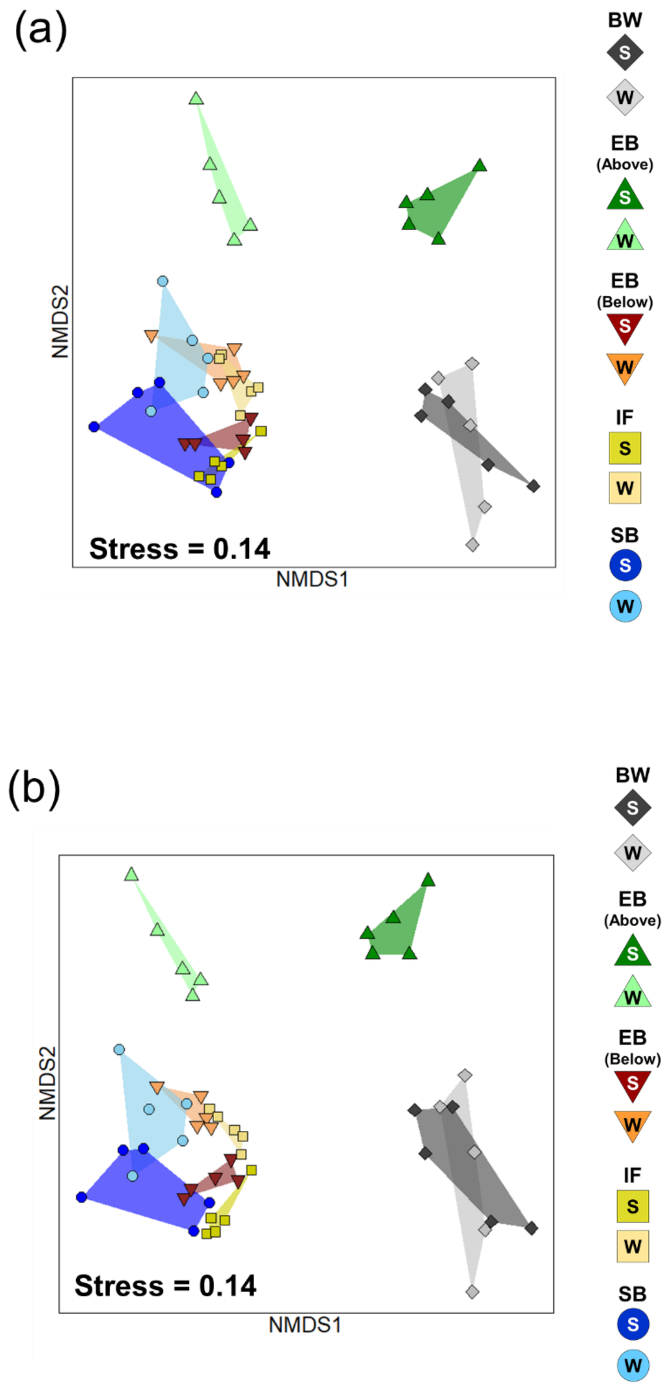

Figure S4. Non-metric multidimensional scaling (NMDS) ordinations of (a) species compositions based on Jaccard dissimilarities calculated from presence/absence data, and (b) species compositions and abundances based on Bray–Curtis dissimilarities calculated

from fourth-root transformed abundance data. The four different symbols indicate subsites in each habitat. Convex hulls enclosed by symbols indicate the dispersion of community composition within habitats. Habitat abbreviations are as shown in Fig. 1.

Supplementary tables

Table S1. Water, sediment, and eelgrass conditions (mean ± standard deviation) at each habitat in summer and winter. ORP: oxidation-reduction potential; D_{50}: median particle size. Habitat abbreviations are as in Fig. 1.

Factor	BW	EB	IF	SB
**Water conditions**				
Summer				
Water temperature (°C)	27.2	27.2 ± 0.3	27.2 ± 0.1	27.2 ± 0.6
Salinity (psu)	30.9	30.7 ± 0.1	30.8 ± 0.1	31.3 ± 0.2
pH	8.1	8.1 ± 0.1	8.1 ± 0.0	8.0 ± 0.1
Dissolved oxygen (mg/L)	6.5	5.3 ± 0.2	6.2 ± 0.4	4.9 ± 1.0
Winter				
Water temperature (°C)	11.9	13.5 ± 0.2	10.5 ± 0.3	12.2 ± 0.7
Salinity (psu)	31.4	30.8 ± 0.1	30.9 ± 0.1	31.3 ± 0.2
pH	8.3	8.3 ± 0.0	8.4 ± 0.1	8.3 ± 0.0
Dissolved oxygen (mg/L)	9.3	8.6 ± 0.2	9.3 ± 0.1	9.2 ± 0.5
**Sediment conditions**				
Summer				
Sediment temperature (°C)	—	26.6 ± 0.6	27.4 ± 0.9	26.7 ± 0.1
pH	—	7.6 ± 0.1	7.6 ± 0.1	7.6 ± 0.0
ORP (mV)	—	-197 ± 39	-189 ± 23	-168 ± 13
Water content (%)	—	52.4 ± 20.3	41.5 ± 20.2	59.7 ± 8.8
D_{50} (mm)	—	0.02 ± 0.01	0.08 ± 0.09	0.01 ± 0.00
Winter				
Sediment temperature (°C)	—	13.1 ± 0.2	9.8 ± 0.4	11.9 ± 1.7
pH	—	7.7 ± 0.1	7.5 ± 0.1	7.4 ± 0.1
ORP (mV)	—	-123 ± 6	-150 ± 60	-167 ± 28
Water content (%)	—	47.7 ± 21.6	43.8 ± 17.4	59.7 ± 18.9
D_{50} (mm)	—	0.02 ± 0.01	0.02 ± 0.01	0.01 ± 0.00

Table S1. *Continued.*

Factor	BW	EB	IF	SB
**Eelgrass conditions**				
Summer				
Shoot density (inds. m^{-2})	—	45.0 ± 17.3	—	—
Shoot mass (wet g m^{-2})	—	132.9 ± 84.5	—	—
Winter				
Shoot density (inds. m^{-2})	—	87.0 ± 20.2	—	—
Shoot mass (wet g m^{-2})	—	5.1 ± 2.7	—	—

Table S2. Species list with abundances and functional traits (PFT: primary feeding type; CLF: common life form) across habitats and seasons. Species marked "(D)" were deemed dominant species for functional composition analysis. Functional groups are abbreviated as follows: **Primary feeding types:** *Ca*, carnivore; *Dp*, deposit feeder; *Dt*, detritus feeder; *FS*, filter/suspension feeder; *Gr,* grazer; *He*, herbivore; *Om*, omnivore; *Sc*, scavenger; *Sy*, symbiont. **Common life forms:** *BU*, burrowing; *FL*, free-living; *PA*, parasite; *SE*, sedentary; *TM*, tube/mucus-sheath building. Habitat abbreviations are as shown in Fig. 1.

Species	SUMMER				WINTER				Functional traits		
	BW	EB	IF	SB	BW	EB	IF	SB	PFT	CLF	Reference
**Polychaeta**											
Ampharetidae sp. (D)			9	6			1	1	Dp	TM	e
Amphinomidae sp. (D)				1					Ca	FL	e
Armandia lanceolata (D)		2	9						Dp	FL	e
Asychis disparidentata		2	1			1					
Branchiomma cingulatum	8	1			4						
Capitellidae sp. (D)	1	27	9	7		11	8	2	Dp	TM	e
Chaetopteridae sp. (D)			1				1	2	Dp	TM	e
Chone sp. (D)		5	1	1		2	28		FS	SE	e
Chrysopetalidae sp.		3	1		29		2				
Cirratulidae sp. (D)	496	1			153		6		Dp	FL	e
Cirriformia tentaculata (D)	100				276				Dp	FL	e

Table S2. *Continued.*

Species	SUMMER				WINTER				Functional traits		
	BW	EB	IF	SB	BW	EB	IF	SB	PFT	CLF	Reference
Dorvilleidae spp.		1			17						
Eteone longa (D)						5	8	2	Ca	FL	e
Eulalia viridis (D)		1			1	14	3	5	Ca	FL	e
Eunice sp.	3										
Eunicidae sp.					2						
Flabelligeridae sp. (D)		1	18	1			1		Dp	TM	e
Glycera chirori (D)		9	10	6		4	6	2	Ca	BU	e
Glycera sp.		2	1			1					
Glyceridae sp.						1					
Goniada sp. (D)						7	52	6	Ca	BU	e
Hemipodia sp. (D)			17			2		1	Ca	BU	e
Hesionidae sp. (D)	15	1	3	3	1	7	10	1	Ca	FL	c; e
Hydroides ezoensis (D)	78				240				FS	SE	e
Hydroides sp.	3										
Lagis bocki (D)		2	5			2	2	2	Dp	TM	e
Lepidonotus elongatus (D)	321				165				Ca	FL	e; h
Lepidonotus tenuisetosus					30						
Lumbrineridae sp.			1		1						
Lysidice collaris	4				4						

Table S2. *Continued.*

Species	SUMMER				WINTER				Functional traits		
	BW	EB	IF	SB	BW	EB	IF	SB	PFT	CLF	Reference
Maldanidae sp. (D)		17				1	1		Dp	TM	e; g; j
Neanthes acuminata (D)		8				9			Om	TM	e
Nectoneanthes oxypoda (D)						24		6	Om	TM	e
Nectoneanthes sp. (D)				1					Om	TM	e
Nephtyidae sp. (D)								1	Ca	FL	e; j
Nephtys sp. (D)		3	5	5		25	45	23	Ca	FL	e; j
Nereiphylla castanea	60				36						
Nereis sp.					10						
Notomastus latericeus (D)		1	8				9	1	Dp	TM	e
Opheliidae sp.						1	10				
Orbiniidae sp.			1			2					
Oxydromus angustifrons (D)			6	1			4		Ca	FL	c; e
Paraonidae sp.		3				1					
Perinereis cultrifera	22				1	1					
Perinereis nuntia brevicirris					1						
Phyllodoce maculata (D)		5				10			Ca	FL	e
Phyllodocidae sp.	2	7			5	3					
Platynereis bicanaliculata (D)		32				3			Om	TM	e
Platynereis sp. (D)	16	104				1			Om	TM	e

Table S2. *Continued.*

Species	SUMMER				WINTER				Functional traits		
	BW	EB	IF	SB	BW	EB	IF	SB	PFT	CLF	Reference
Poecilochaetidae sp. (D)		1	1	3			2	1	FS	TM	e
Polynoidae sp.	49	3	1		17	3	6				
Polyophthalmus pictus		3			1						
Praxillella affinis pacifica (D)		19	75	30		3	21		Dp	TM	e; g; j
Prionospio pulchra						1					
Prionospio sp. (D)		1				9	1	6	Dp	TM	e
Pseudopolydora kempi (D)						50			Dp	TM	e
Pseudopolydora paucibranchiata (D)						64			Dp	TM	e
Pseudopolydora sp. (D)	254	2	4		613	14	148	17	Dp	TM	e
Pseudopotamilla sp. (D)	743				932				FS	SE	e
Sabellariidae sp.							1				
Sabellida sp.		10									
Sabellidae sp. (D)	4	6			190	5	7	1	FS	SE	e
Scolelepis sp. (D)	1	2	3	1		3	1		Dp	TM	e
Scoletoma longifolia (D)		168	38	20		127	38	5	Ca	BU	e
Serpulidae sp.	9				20						
Sigalionidae sp. (D)		4	2	9		13	4	16	Ca	FL	e
Sigambra sp. (D)		3		3		5	21	9	Om	FL	c; e; h; j
Simplisetia erythraeensis (D)			19			2	1		Om	TM	e
Spionidae sp.		4									

Table S2. *Continued.*

Species	SUMMER				WINTER				Functional traits		
	BW	EB	IF	SB	BW	EB	IF	SB	PFT	CLF	Reference
Spirobranchus kraussii (D)	1157				1584				FS	SE	e
Spirobranchus tetraceros	3				5						
Sternaspis scutata (D)				10		43		1	Dp	BU	j; p (cf. Terebellida)
Syllidae sp. (D)	5	8			144				Ca	TM	c; e
Syllis sp. (D)	535					9	1	6	Dp	TM	e
Terebellidae sp. (D)	834	2			879	1			Dp	TM	e
Thelepus setosus					28						
**Amphipoda**											
Ampithoe sp.	3				9						
Ampithoidae sp.		4									
Aoridae sp.		5									
Aoroides columbiae (D)						155	9	2	Om	TM	c; n; p
Byblis japonicus		3				3					
Caprella sp. (D)		6			8	4			FS	FL	c; n; p
Corophiidae sp. (D)						1		1	FS	TM	c; n
Corophium sp. (D)			2				210		FS	TM	c; n
Dexaminidae sp.		2									
Ericthonius pugnax (D)	3	1						2	FS	TM	c; p
Grandidierella japonica							9				
Hyale sp.	1				9						

Table S2. *Continued.*

Species	SUMMER				WINTER				Functional traits		
	BW	EB	IF	SB	BW	EB	IF	SB	PFT	CLF	Reference
Leucothoidae sp.					6						
Monocorophium acherusicum (D)	145				3071				FS	TM	c; n
Nippopisella nagatai (D)			34	1			6		He	FL	c; p (cf. Hadzioidea)
Oedicerotidae sp.						2					
**Cumacea**											
Dimorphostylis sp.						1	1				
**Isopoda**											
Dynoides dentisinus	3				1						
Gnorimosphaeroma rayi (D)	3				192	1			Om	FL	m
Ianiropsis longiantennata					4						
Ligia sp.	6										
Munnidae sp.					32						
Paranthuridae sp.		7									
Sphaeroma sieboldii	168				137						
**Tanaidacea**											
Zeuxo normani (D)		307							FS	TM	c; d (cf. Tanaidacea)
**Ostracoda**											
Myodocopina sp.		11			7	1					
Podocopida sp. (D)		32				1			Om	FL	c; p
Vargula hilgendorfii (D)		7	1			3			Om	FL	c; p

Table S2. *Continued.*

Species	SUMMER				WINTER				Functional traits		
	BW	EB	IF	SB	BW	EB	IF	SB	PFT	CLF	Reference
**Hexanauplia**											
Harpacticoida sp.						5					
Porcellidiidae sp. (D)		550							Dt; Gr	FL	c; p
**Decapoda**											
Alpheidae sp.	4	2									
Actaea sp.	1										
Alpheus brevicristatus								1			
Alpheus sp. (D)			7	2	3				Om	FL	k; p (cf. Caridea)
Athanas sp. (D)		4	8	1			1		Om	FL	k; p (cf. Caridea)
Camptandrium sexdentatum (D)		2	31	5		1	4		Om	BU	k; p (cf. Pleocyemata)
Charybdis japonica	1										
Charybdis sp.			1								
Decapoda sp.		2									
Hayashidonus japonicus		1									
Hexapinus sp. (D)				2					Om	PA	i; p (cf. Pleocyemata)
Hexapodidae sp.				1			1				
Latreutes laminirostris		1									
Latreutes planirostris						1					
Latreutes sp.					1						
Macromedaeus distinguendus	154				134						

Table S2. *Continued.*

Species	SUMMER				WINTER				Functional traits		
	BW	EB	IF	SB	BW	EB	IF	SB	PFT	CLF	Reference
Macrophthalmus sp.							6				
Majidae sp.	1	2									
Nanosesarma minutum (D)	216				263				Om	FL	k; p (cf. Pleocyemata)
Ocypodidae sp. (D)				1					Om	BU	k; p (cf. Pleocyemata)
Ogyrides striaticauda (D)			8				6		Om	FL	p (cf. Caridea)
Paguridae sp.	8				2						
Petrolisthes sp. (D)	254				69				FS	FL	c; k
Pilumnopeus makianus	9										
Pilumnus minutus	1				23						
Pinnotheres sp.						1					
Pinnotheridae sp. (D)		4	1	2					Om	PA	c; k; p (cf. Pleocyemata)
Porcellanidae sp.			1								
Portunus sp.			1								
Pseudopinnixa carinata							1				
Raphidopus ciliatus (D)			1	4					FS	FL	c; k
Sphaerozius nitidus					1						
Thalamita sima	1										
Typhlocarcinus sp.			1								
Upogebia major						1					
Xanthidae sp. (D)	92				98				Om	FL	k; p (cf. Pleocyemata)
Xenophthalmus pinnotheroides (D)		3	2	2		1	1	1	Om	FL	a; p (cf. Pleocyemata)

Table S2. *Continued.*

Species	SUMMER				WINTER				Functional traits		
	BW	EB	IF	SB	BW	EB	IF	SB	PFT	CLF	Reference
**Stomatopoda**											
Oratosquilla oratoria								1			
**Sessilia**											
Amphibalanus amphitrite (D)	2422		1		1586				FS	SE	c; k; p (cf. Balanomorpha)
Amphibalanus eburneus	56				2						
Amphibalanus reticulatus (D)	67		136		12				FS	SE	c; k; p (cf. Balanomorpha)
Balanus trigonus (D)	2121				1001				FS	SE	c; k; p (cf. Balanomorpha)
Chthamalus challengeri	128				36						
Fistulobalanus albicostatus (D)	1123				1374				FS	SE	c; k; p (cf. Balanomorpha)
**Collembola**											
Collembola sp.					1						
**Insecta**											
Chironomidae sp. (D)					72				Om	FL	b; c; f; o
Tipulidae sp.					76						
**Gastropoda**											
Aplysiidae sp.		8									
Cerithiidae sp.					46						
Alaba sp. (D)		36							Dt	FL	c (cf. Cerithioidea); l
Alvania concinna		10			2						
Bedevina birileffi (D)	18	3		2	60				Ca-Sc	FL	c; l; p

Table S2. *Continued.*

Species	SUMMER				WINTER				Functional traits		
	BW	EB	IF	SB	BW	EB	IF	SB	PFT	CLF	Reference
Cantharidus japonicus		2									
Cellana toreuma	1										
Columbellidae sp.		9									
Cylichnatys angusta (D)							121		Gr	FL	c; l; p
Decorifer insignis (D)							62		Ca	FL	c; l; p
Diala semistriata		6			1						
Drupella margariticola (D)						4		1	Ca-Sc	FL	c; l; p
Elysia sp.					2						
Erato sp.	1	1									
Ergalatax sp.	1										
Gastropoda sp.		17									
Haminoeidae sp. (D)		1				4			Gr	FL	c; l; p
Lacuna sp.						1					
Littoraria articulata					16						
Littoraria sp.	1										
Littorina brevicula (D)	145				188				Gr	FL	c; l
Mitrella bicincta	1										
Mitrella sp.		5									
Muricidae sp.	1				9						
Nassarius livescens (D)		9		19		5		1	Sc	FL	c; p

Table S2. *Continued.*

Species	SUMMER				WINTER				Functional traits		
	BW	EB	IF	SB	BW	EB	IF	SB	PFT	CLF	Reference
Nassarius multigranosus						7					
Nassarius praematuratus						2					
Nassarius sp.		6	2								
Notocochlis gualtieriana							1				
Nudibranchia sp.						1					
Onchidiidae sp.					1						
Peasiella habei	160				100						
Philine orientalis			1								
Pyramidellidae sp.	1				88						
Reishia bronni					1						
Reishia clavigera	10				2						
Reticunassa festiva (D)	3	8	6	11		8	14	4	Sc	FL	c; p
Reticunassa sp.						1	1				
Rissoidae sp.		19									
Siphonaria japonica					2						
Stenothyra edogawensis							1				
Thylacodes adamsii	51				98						
Triphoridae sp.					1						
Yokoyamaia ornatissima (D)				7					Ca	FL	c; l; p
Zafra mitriformis (D)	20				134				Ca-Sc	FL	c; l; p
Zafra sp.		8				1					

Table S2. *Continued.*

Species	SUMMER				WINTER				Functional traits		
	BW	EB	IF	SB	BW	EB	IF	SB	PFT	CLF	Reference
**Bivalvia**											
Anomia Chinensis	1				1						
Arcuatula senhousia (D)	27	673	118	3	5	28	716	4	FS	SE	c; l
Bivalvia sp.		9									
Chama japonica	2				3						
Crassostrea gigas	19										
Cyclina sinensis							2				
Fulvia hungerfordi			1			3	3	2			
Irus irus	1										
Irus sp.	2				1						
Lasaea undulata (D)	1015				644				FS	BU	c; l; p (cf. Galeommatoidea)
Laternula gracilis							10				
Leptomya minuta						1					
Leucosphaera oyamai						1					
Lima sp.	1										
Lutraria sieboldii							4				
Macoma incongrua							2				
Macoma sp.							2				
Moerella rutila (D)			14				83		FS-Dp	BU	c; l; p
Mytilidae sp.					1						

Table S2. *Continued.*

Species	SUMMER				WINTER				Functional traits		
	BW	EB	IF	SB	BW	EB	IF	SB	PFT	CLF	Reference
Nitidotellina lischkei (D)		2	2			1	3		FS-Dp	BU	c; l; p
Ostreidae sp.					109						
Paratapes undulatus (D)				3					FS	BU-SE	c; l
Petricola japonica					10						
Petricola sp.	12										
Ruditapes philippinarum (D)			9				8		FS	BU	c
Solemya pusilla (D)		19		2					Sy	BU-SE	l; p
Solen brevissimus				1							
Theora lata (D)		2	1			91	35	88	Dp	BU	c; l; p
Thraciidae sp. (D)		1	1	10					FS	BU	c (cf. Bivalvia); p
Timoclea micra						1					
Xenostrobus atratus (D)	577				740				FS	BU	c; l
**Polyplacophora**											
Acanthochitona achates	35				18						
Liolophura japonica					1						
Ischnochitonidae sp.	1										
**Brachiopoda**											
Discradisca sp.	7				9						
**Echinodermata**											
Temnopleurus toreumaticus	3										

Table S2. *Continued.*

Species	SUMMER				WINTER				Functional traits		
	BW	EB	IF	SB	BW	EB	IF	SB	PFT	CLF	Reference
Amphioplus (Lymanella) japonicus						1					
Amphiura (Ophiopeltis) aestuarii							2				
Amphiuridae sp. (D)		3		2				1	Dt	FL	c; k
Ophiothrix sp.					4						
Ophiurida sp.		1				1	14				
Ophiuroidea sp.	2										
Synaptidae sp. (D)		6	10	4		2	3	1	Dt	BU	c (cf. Holothuroidea); p
**Bryozoa**											
Watersipora cucullata	1	1			15						
**Nemertea**											
Nemertea sp. (D)	5	4	6	5	78	4	5	4	Ca	FL	c; j
**Sipuncula**											
Sipuncula sp.							1	1			
**Actiniaria**											
Actiniaria sp.	13	3					1				
Diadumene lineata					8						
**Ascidiacea**											
Ascidiidae sp.					2						
Polyandrocarpa sp.					5						
Pyuridae sp.	2				5						

Table S2. *Continued.*

Species	SUMMER				WINTER				Functional traits		
	BW	EB	IF	SB	BW	EB	IF	SB	PFT	CLF	Reference
Styela plicata	1										
Styelidae sp.	1										
**Chaetognatha**											
Sagittoidea sp.						3					
**Platyhelminthes**											
Platyhelminthes sp. (D)	112	68			144				Ca	FL	c
**Porifera**											
Halichondria (Halichondria) japonica					6						
Halichondria (Halichondria) okadai					27						

References for classification of functional groups

a. Aramaki, Y., Taguchi, K., Hishiki, K., Suda, Y., Murai, T. 2005. Crabs appeared in tidal flat of Okinohata, Yanagawa, Fukuoka prefecture [Japan] and some ecological findings of *Xenophthalmus pinnotheroides* (in Japanese with English abstract). *Journal of National Fisheries University*, 53: 9-19.

b. Brodin, Y., Andersson, M. H. 2009. The marine splash midge *Telmatogon japonicus* (Diptera; Chironomidae)—extreme and alien? *Biological Invasions*, 11: 1311-1317.

c. Carlton, J. T. (ed.). 2007. The light and Smith manual: intertidal invertebrates from Central California to Oregon. 4th edition. *Berkeley: University of California Press*.

d. The Encyclopedia of Life. https://eol.org/.

e. Fauchald, K., Jumars, P. A. 1979. The diet of worms: a study of polychaete feeding guilds. *Oceanography and Marine Biology: An Annual Review*, 17: 193-284.

f. Henriques-Oliveira, A. L., Nessimian, J. L., Dorvillé, L. F. M. 2003. Feeding habits of chironomid larvae (Insecta: Diptera) from a stream in the Floresta da Tijuca, Rio de Janeiro, *Brazil. Brazilian Journal of Biology*, 63: 269-281.

g. ImajimaM (ed.). 1996. Polychaetous Annelids. *Tokyo: Aquabiology*.

h. ImajimaM (ed.). 2001. Polychaetous Annelids II. *Tokyo: Aquabiology*.

Table S2. *Continued.*

References for classification of functional groups	
**i.**	Naruse, T., Watanabe, T., Yoshida, R. 2017. Additional information on distribution and habitats of six hexapodid species (Crustacea: Decapoda: Brachyura), with review of their Japanese names. *Fauna Ryukyuana*, 35: 17-28.
**j.**	Nishimura S (ed.). 1992. Guide to seashore animals of Japan with color pictures and keys (in Japanese), vol. 1. *Osaka: Hoikusha*.
**k.**	Nishimura S (ed.). 1995. Guide to seashore animals of Japan with color pictures and keys (in Japanese), vol. 2. *Osaka: Hoikusha*.
**l.**	Okutani T. 2000. Marine mollusks in Japan. *Tokyo: University of Tokyo Press*.
**m.**	Poore, G. C., Bruce, N. L. 2012. Global diversity of marine isopods (except Asellota and crustacean symbionts). *PLoS One*, 7: e43529.
**n.**	Scipione, M. B. 2013. Do studies of functional groups give more insight to amphipod biodiversity? *Crustaceana*, 86: 955-1006.
**o.**	Silva, F.L., Ruiz, S.S., Bochini, G.L., Moreira, D.C. 2008. Functional feeding habitats of Chironomidae larvae (Insecta, Diptera) in a lotic system from Midwestern region of São Paulo State, Brazil. *Pan-American Journal of Aquatic Sciences*, 3: 135-141.
**p.**	World Register of Marine Species (WoRMS). http://www.marinespecies.org/.
